# Craniofacial-specific transcriptomics uncovers novel genes underlying jaw divergence in dietary specialist pupfishes

**DOI:** 10.1093/genetics/iyaf207

**Published:** 2025-09-26

**Authors:** M Fernanda Palominos, Vanessa Muhl, Christopher H Martin

**Affiliations:** Department of Integrative Biology, University of California, Berkeley, Berkeley, CA 94720, United States; Museum of Vertebrate Zoology, University of California, Berkeley, Berkeley, CA 94720, United States; Department of Integrative Biology, University of California, Berkeley, Berkeley, CA 94720, United States; Museum of Vertebrate Zoology, University of California, Berkeley, Berkeley, CA 94720, United States; Department of Integrative Biology, University of California, Berkeley, Berkeley, CA 94720, United States; Museum of Vertebrate Zoology, University of California, Berkeley, Berkeley, CA 94720, United States

**Keywords:** evo-devo, tissue-specific, transcriptomics, adaptive radiation, craniofacial development, speciation, gene expression

## Abstract

Changes in gene expression underlie most phenotypic differences among closely related species. While previous studies in model systems have identified conserved genes and pathways involved in craniofacial evolution, less is known about gene expression differences associated with craniofacial divergence in rapidly evolving species. Here, we investigate craniofacial-specific gene expression in a nascent adaptive radiation of *Cyprinodon* pupfishes endemic to San Salvador Island, Bahamas, which includes 3 trophic specialists with highly divergent craniofacial morphologies (two scale-eaters and a molluscivore) derived from an ancestral Caribbean-wide generalist. We compared gene expression in the most morphologically divergent craniofacial region with the relatively conserved caudal region across 5 *Cyprinodon* species and 9 populations. We focused on the hatchling stage, the earliest developmental stage at which craniofacial differences among species are evident. Our approach revealed a large proportion of differentially expressed genes (DEGs) found exclusively in the craniofacial region of the specialists only. By intersecting these specialist-specific craniofacial-exclusive genes with genomic regions harboring fixed single-nucleotide variants under selection in the specialists, we identified 14 candidate genes. We confirmed novel craniofacial expression for 2 of these candidates, *pycr3* and *atp8a1*, genes not previously associated with craniofacial development or function, in hatchlings using in situ mRNA hybridization and observed species-specific differences in the pharyngeal arches and craniofacial muscles, respectively. Our findings demonstrate how an “evolutionary mutant” model can reveal novel gene expression patterns, highlighting the power of integrating tissue-species transcriptomics with speciation genomics to identify novel regulators of craniofacial evolution.

## Introduction

Adaptive radiations inform our understanding of the genetic bases of phenotypic evolution in vertebrates (Powder and Albertson 2016; Martin and Richards 2019; Gillespie et al. 2020). Young adaptive radiations, in particular, provide a framework for studying how changes in gene expression lead to phenotypic divergence (Schluter 2000; Martin et al. 2019). Perhaps one of the most famous examples is the Galápagos radiation of Darwin's finches, in which *bmp4* (*bone morphogenetic protein 4*) levels and timing of expression vary among finch species during craniofacial development (Abzhanov et al. 2004) and variants in the coding and noncoding regions of *alx1* (*Alx homeobox 1*) control differences in beak size and sharpness (Lamichhaney et al. 2015; Rubin et al. 2022). Studies of the immense craniofacial diversity in adaptive radiations of fishes have shown that divergent craniofacial traits often arise from the expansion or reduction of the expression field of known craniofacial genes during embryonic or postembryonic development. For example, a genetic substitution in the enhancer region of *bmp6* (*bone morphogenetic protein 6*) increases tooth number in freshwater sticklebacks relative to marine populations by regulating the size of tooth-forming fields (Cleves et al. 2014, 2018; Stepaniak et al. 2021); similarly, in Lake Malawi cichlids, craniofacial morphological divergence is linked to changes in expression of other known craniofacial genes, such as *bmp4* (Albertson et al. 2005; Powder et al. 2014), *fgf* (Albertson et al. 2018), *lbh* (Powder et al. 2014), *ptch1* (Roberts et al. 2011; Hu and Albertson 2014; DeLorenzo et al. 2022), and *sox9b* (Matthews and Albertson 2017), among others (Streelman et al. 2003; Conith and Albertson 2021).

Despite advances in understanding how well-known craniofacial genes contribute to the evolution of divergent morphologies among radiating species, the potential of adaptive radiations for providing insights into novel pathways affecting human craniofacial variation and congenital disorders (Naqvi et al. 2022) has been largely overlooked (Gross and Powers 2020; Powers et al. 2023). Importantly, the discovery of novel gene networks behind the craniofacial variance in natural systems provides insights into rare and small-effect human craniofacial genetic syndromes (Streelman et al. 2007; Albertson et al. 2009; Powder and Albertson 2016).

The San Salvador Island (SSI) *Cyprinodon* adaptive radiation is comprised of 4 pupfish species occurring in sympatry across several hypersaline lakes (Martin and Wainwright 2013a, 2013b), including 3 endemic trophic specialists: the molluscivore pupfish (*C. brontotheroides*) with a novel nasal and maxillary skeletal protrusion; the specialized scale-eating pupfish (*C. desquamator*) with greatly enlarged oral jaws and adductor mandibulae (AM) muscles (Hernandez et al. 2018); and a recently discovered intermediate scale-eater (*Cyprinodon* sp. “wide-mouth”), with the widest oral jaws and intermediate jaw lengths (Richards and Martin 2022). Although both *C. desquamator* and *Cyprinodon* sp. “wide-mouth” are scale-eaters, they exhibit distinct craniofacial morphologies, behaviors, and genetic signatures of selection (Richards et al. 2021; Richards and Martin 2022). There is intraspecific morphological variation among lakes (Martin and Feinstein 2014), but the most pronounced craniofacial differences observed are among species irrespective of their source lake population. All specialist pupfishes in SSI are mostly closely related to the Caribbean-wide generalist species (*C. variegatus*) also present in SSI's lakes. Originating approximately 10,000 years ago, the SSI pupfish radiation rapidly evolved divergent and unique craniofacial phenotypes despite minimal genetic divergence (Martin and Wainwright 2011; Martin et al. 2017; McGirr and Martin 2017, 2021) and ongoing gene flow (Richards and Martin 2017; Richards et al. 2021; Patton et al. 2022). Notably, the strikingly different craniofacial morphologies of each pupfish species are apparent as early as hatching (Holtmeier 2001; Lencer et al. 2016; Lencer and McCune 2020; Palominos et al. 2023) and remain highly divergent after multiple generations of laboratory rearing in a common garden environment on a common diet. Combined with their amenability to laboratory rearing, this system is excellent for studying how genetic changes lead to extreme craniofacial differences among closely related species. Previously, we discovered a novel function for *galr2* (*galanin receptor 2*) in craniofacial development in lower oral jaw elongation in the scale-eating pupfish (Palominos et al. 2023), highlighting the potential of this system to reveal novel gene function in craniofacial development.

Gene expression regulation is likely the dominant mechanism driving phenotypic diversity among closely related species (Wray 2007; Uebbing et al. 2016; Carruthers et al. 2022; Duenser et al. 2024). Previous transcriptomic studies in SSI pupfishes identified differentially expressed genes (DEGs) between species in canonical craniofacial developmental pathways such as IgF (*insulin-like growth factor*) and Wnt (*wingless-related integration site*) (Lencer et al. 2017), as well as cell cycle regulation (Lencer and McCune 2020). Previous work from our lab linked genetic variants with gene expression changes in whole embryos and larvae and identified 2 novel craniofacial candidate genes *dync2li1* and *pycr3*, differentially expressed from embryonic through post-larval development (McGirr and Martin 2021). However, DEGs in pupfishes have not yet been studied within specific tissues, nor included outgroup generalist pupfish species for comparison.

Substantial morphological analyses have demonstrated that the craniofacial region, particularly the oral jaws, exhibits the fastest rate of morphological diversification on SSI relative to generalist pupfish populations on neighboring islands, while the caudal region shows a low relative rate of diversification across all pupfishes (Martin and Wainwright 2011, 2013b; Martin 2016; Martin and Gould 2020). In addition to different embryonic origins of neural crest for craniofacial structures and somitic mesoderm for the caudal region, they also differ in ecological relevance; jaw morphology directly affects dietary specialization while the caudal region and caudal fin affect locomotion.

We used high-coverage RNA sequencing of craniofacial and caudal tissues to contrast a rapidly evolving and functionally divergent morphological region with a slowly evolving morphological region. By including 4 pupfish species within the SSI radiation (generalist, molluscivore, scale-eater, and “wide-mouth”) and 2 generalist outgroup species (*C. fontinalis* from Chihuahua, Mexico, and an outgroup population of *C. variegatus* from North Carolina), we identified and validated 2 novel craniofacial-specific DEGs. Our design captured gene expression patterns underlying phenotypic divergence during embryonic development and linked them to fixed genetic variants unique to SSI trophic specialists.

## Materials and methods

### Study system and pupfish husbandry

*Cyprinodon* species were maintained in the lab for at least 2 generations in small breeding groups. Two lake populations of scale-eaters (*C. desquamator*), 2 lake populations of molluscivores (*C. brontotheroides*), 1 population of intermediate scale-eater (*Cyprinodon* sp. “wide-mouth”), and 4 populations of generalists (*C. variegatus*, *C. fontinalis*) were reared in the lab for multiple generations and sampled for tissue-specific gene expression at hatching (at 8 d post-fertilization). SSI *C. variegatus* and *C. brontotheroides* were collected from Osprey Lake and Crescent Pond in 2018; *C. desquamator* individuals were collected from Crescent Pond in 2018 and Little Lake in 2014 (which is connected to Osprey Lake through the large interior Great Lake system). A fourth sympatric and newly described intermediate scale-eater pupfish, *Cyprinodon* sp. “wide-mouth,” was collected from Osprey Lake in 2018 (Richards and Martin 2022). We also included 2 outgroup generalist populations: *C. variegatus* from North Carolina collected in 2018 and a more distant outgroup *C. fontinalis* from Ojo de Carbonera in the Chihuahuan Desert in Mexico obtained from the American Killifish Association captive breeding program in 2020. These outgroups represent approximately 10,000 and 30,000 years of divergence from the SSI species flock, respectively (Martin 2016; Martin et al. 2016; Tian et al. 2022).

### Craniofacial and caudal region sample collection

**Fig. 1.**
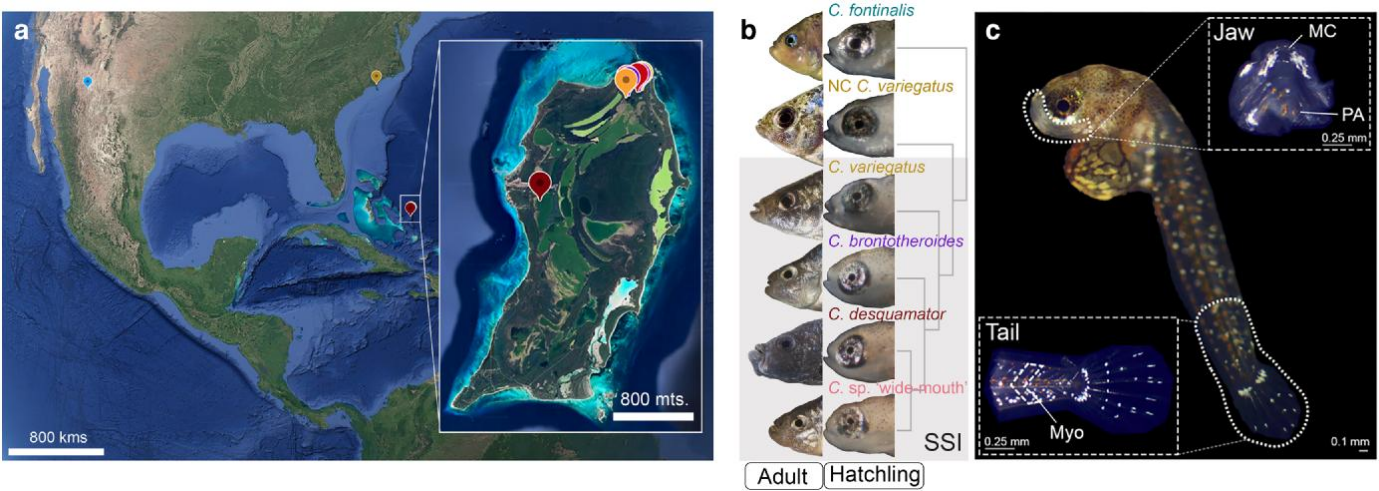
Comparative craniofacial and caudal region-specific transcriptomics among San Salvador Island (SSI; grey shaded box) pupfishes and outgroup generalist species at hatching. The craniofacial and caudal region-specific RNA samples were isolated from 5 lab-reared pupfish species originating from 5 different sample locations. a) We included 3 populations of the generalist pupfish *C. variegatus*: Crescent Pond (yellow) and Osprey Lake (orange) in San Salvador Island, and *C. variegatus ovinus* from Fort Fisher, North Carolina (NC, mustard brown), United States. *C. fontinalis* (blue), also a generalist, is endemic to a single oasis in the Chihuahua desert in Mexico separated by approximately 100,000 years of divergence from the San Salvador Island radiation and the NC generalist (Tian et al. 2022). *C. brontotheroides* was collected from Crescent Pond and Osprey Lake, *C. desquamator* was collected from Crescent Pond and Little Lake (dark red), and *C*. sp. “wide-mouth” was collected from Osprey Lake. Inset: San Salvador Island. Crescent Pond and Osprey Lake in SSI are adjacent but separated by a 20 m ridge. Satellite images from Google Earth. b) Adult and hatchling craniofacial morphology and evolutionary relation (not to scale). San Salvador Island pupfish species are inside the orange rectangle. c) Top inset: the craniofacial tissue dissection included the oral jaws (dentary, angular articular), maxilla, premaxilla, palatine, the pharyngeal jaws, frontonasal region, and the surrounding connective tissue, epithelia, and muscle. Bottom inset: the caudal tissue dissection included the caudal tail, the first 6 myomere from posterior to anterior, notochord, and developing rays of the caudal fin.

Embryos were collected within 24 h after natural fertilization in mixed breeding tanks and moved to a Petri dish for constant incubation at 27 °C in 2–5 ppt salinity dechlorinated water with a diluted addition of methylene blue and gentamycin to prevent fungus. Newly hatched larvae from all species and populations were collected in 1 ml sterile microcentrifuge tubes filled with the RNA stabilizer RNA*later* (Thermo Fisher Scientific, Inc.) and stored for 24 h at 4 °C, followed by long-term storage at −20 °C. Under a stereomicroscope, we dissected the oral jaws and associated craniofacial connective tissues, including the dentary, angular articular, maxilla, premaxilla, palatine, pharyngeal apparatus, and frontonasal region from each embryo (Fig. 1c). Five to 6 embryos were pooled per biological replicate to obtain sufficient extracted RNA for sequencing libraries. We similarly dissected the caudal region from each embryo starting from the last 6 myotomes from posterior to anterior, with the caudal fin included (Fig. 1). Petri dishes, spring scissors, and forceps used for dissection were washed with RNAse AWAY (Thermo Fisher Scientific, Inc.) before use. The dissections were carried out in a sterile 30 mm plastic Petri dish filled with RNA*later* using 2 mm fine Spring scissors and fine Dumont forceps (Fine Science Tools, Inc.).

### RNA isolation, sequencing, and alignment

RNA biological replicates were from craniofacial or caudal region tissues. Tissue-specific RNA (see Supplementary Fig. 1) from each of the biological replicates (for each species, population, and tissue) was extracted using Monarch Total RNA Miniprep Kit (New England Biolabs), following the manufacturer's instructions. We validated RNA integrity using an Agilent Bioanalyzer, and only samples with a RIN score > 8 were used for library preparation. The poly-A-enriched libraries and sequencing were carried out by the DNA Technologies and Expression Analysis Core at the UC Davis Genome Center, supported by NIH Shared Instrumentation Grant 1S10OD010786-01. All sequenced samples are listed in Supplementary Table 1, with the respective numbers of technical replicates. Each technical replicate consisted of a sequenced mRNA sample composed of either the dissected craniofacial or caudal tissues pooled from 5 to 6 hatched larvae at 8 dpf.

We used 150 bp paired-end sequencing on 2 lanes of Illumina NovaSeq 6000 S4, resulting in 7.6 billion raw reads. We filtered raw reads using Trim Galore (v. 4.4, Babraham Bioinformatics) to remove Illumina adaptors and low-quality reads (mean Phred score < 20). We mapped reads of all species to the *C. variegatus* reference genome (C_variegatus-1.0, GCA_000732505.1) using the RNA-seq aligner STAR (v. 2.7.10b, parameters = -outFilterMultimapNmax 1, -outFilterMismatchNmax 3). After alignment, reads were counted using HTseq (Anders et al. 2015). Mapping and read quality were assessed using MultiQC (Ewels et al. 2016) (see Supplementary Fig. 1 and Supplementary Table 2 for details).

### Differential expression analyses

We normalized counts and performed differential gene expression analysis using DESeq2 (v.1.40.2; Love et al. 2014) in RStudio [v. 2023.09.1 + 494 (Racine 2012)]. Gene IDs obtained from the Ensembl database (Harrison et al. 2024). Genes with a mean read count greater than ten across samples were retained for analyses, resulting in a set of 20,385 and 18,391 genes (mean across all species) expressed in our craniofacial and caudal region datasets, respectively (Supplementary Table 3). We compared gene expression (i) between each species against all other species in our dataset and (ii) between tissues within each species. For each tissue, we constructed a DESeq2 dataset using a design formula based on a grouping variable (“pooling factor”) that combined species and population identity, enabling us to group biological replicates within each species across lakes, unless otherwise specified. To detect species-specific differentially expressed genes (DEGs), we compared each species against all other species in the dataset using the model: design = ∼ pooling_factor, where pooling_factor indicates the species–population combination (eg *C. desquamator* from Crescent Pond vs. *C. desquamator* from Little Lake). We then extracted pairwise contrasts between focal species and the pooled group of all other species. Differential expression between comparisons was determined using Wald tests of the normalized posterior log fold-change estimates and corrected for multiple testing using the Benjamini–Hochberg method with a false discovery rate (FDR) of 0.01.

To categorize the potential function of craniofacial-exclusive DEGs in each pupfish species comparison, we used gene ontology (GO) enrichment analyses using a pool of 56 out of 96 annotated genes common across the craniofacial replicates of different pupfish species; 1,268 out of 1,963 for *C. brontotheroides*; 3,368 out of 5,159 for *Cyprinodon* sp. “wide-mouth”; and 588 out of 1,070 for *C. desquamator*. We analyzed our species- and craniofacial-specific DEGs with ShinyGo v. 0.77 (Ge et al. 2020), via PANTHER (Mi et al. 2017) and Metascape (Zhou et al. 2019) using annotations available for zebrafish.

To assess differential expression of the overlapping genes between craniofacial-exclusive DEGs and adaptive alleles across species, we normalized and log-transformed gene expression values. We fit linear models using species as a fixed effect. Post hoc pairwise comparisons among species were performed using estimated marginal means (EMMs) implemented in the R emmeans package. *P*-values were adjusted for multiple comparisons using Tukey's honest significant difference (HSD) method. To facilitate interpretation of statistically indistinguishable groups, we used compact letter displays in which groups sharing letters do not differ significantly at *α* = 0.05.

### In situ mRNA hybridization

We used chromogenic ISH for *pycr3* in cryosections using RNAscope 2.5 (Advanced Cell Diagnostics, Inc.) performed by the UNC Translational Pathology and Histopathology Core Service. Moreover, 8 dpf formalin-fixed and paraffin-embedded embryos were sectioned and hybridized to a custom pupfish RNAscope probe on the Leica Biosystems’ Bond RX fully automated system with the RNAscope 2.5 LS BROWN assay kit. Fluorescent ISH for *atp8a1* and *tpm3b* was performed for all species at 8 dpf using hybridization chain reaction (HCR) custom probes from Molecular Instruments, Inc. following the (Palominos et al. 2023) protocol. Samples were imaged using a Zeiss LSM880 (inverted) confocal microscope at the Biological Imaging Facility at the University of California, Berkeley, following Palominos et al. 2023.

## Results and discussion

### Gene expression divergence between craniofacial and caudal regions, species, and populations

To investigate the tissue- and species-specific transcriptional divergence underlying the strikingly different craniofacial morphologies exhibited by SSI pupfishes (Fig. 1), we conducted bulk RNA sequencing on isolated craniofacial (Fig. 1c, top inset) and caudal tissues (Fig. 1c, bottom inset) from hatched larvae at 8 dpf (grown at 27 °C for all species analyzed). Dissections of the craniofacial tissue included the oral jaws (dentary, angular, articular), maxilla, premaxilla, palatine, pharyngeal jaws, and surrounding connective tissue, epithelia, muscle, nerves, and sensory cells among other cell types (Fig. 1c). The dissected caudal region included the caudal fin, the first 6 myomeres from posterior to anterior (also known as the caudal peduncle), notochord, and developing rays of the caudal fin (Fig. 1c). Both the craniofacial and the caudal region dissections also included skin chromatophores.

We sampled the craniofacial and caudal regions across pupfishes within SSI (2 populations per generalist and specialists), 1 outgroup generalist population from North Carolina (NC *C. variegatus*), and a distant outgroup *C. fontinalis* from the Chihuahua desert with at least 100,000 years of divergence from the San Salvador Island species (Fig. 1b and c; Echelle et al. 2005). Our design allowed us to capture transcriptional variation associated with craniofacial evolution among pupfishes and the transcriptional variance associated with the dietary specialization within species occurring on SSI.

**Fig. 2.**
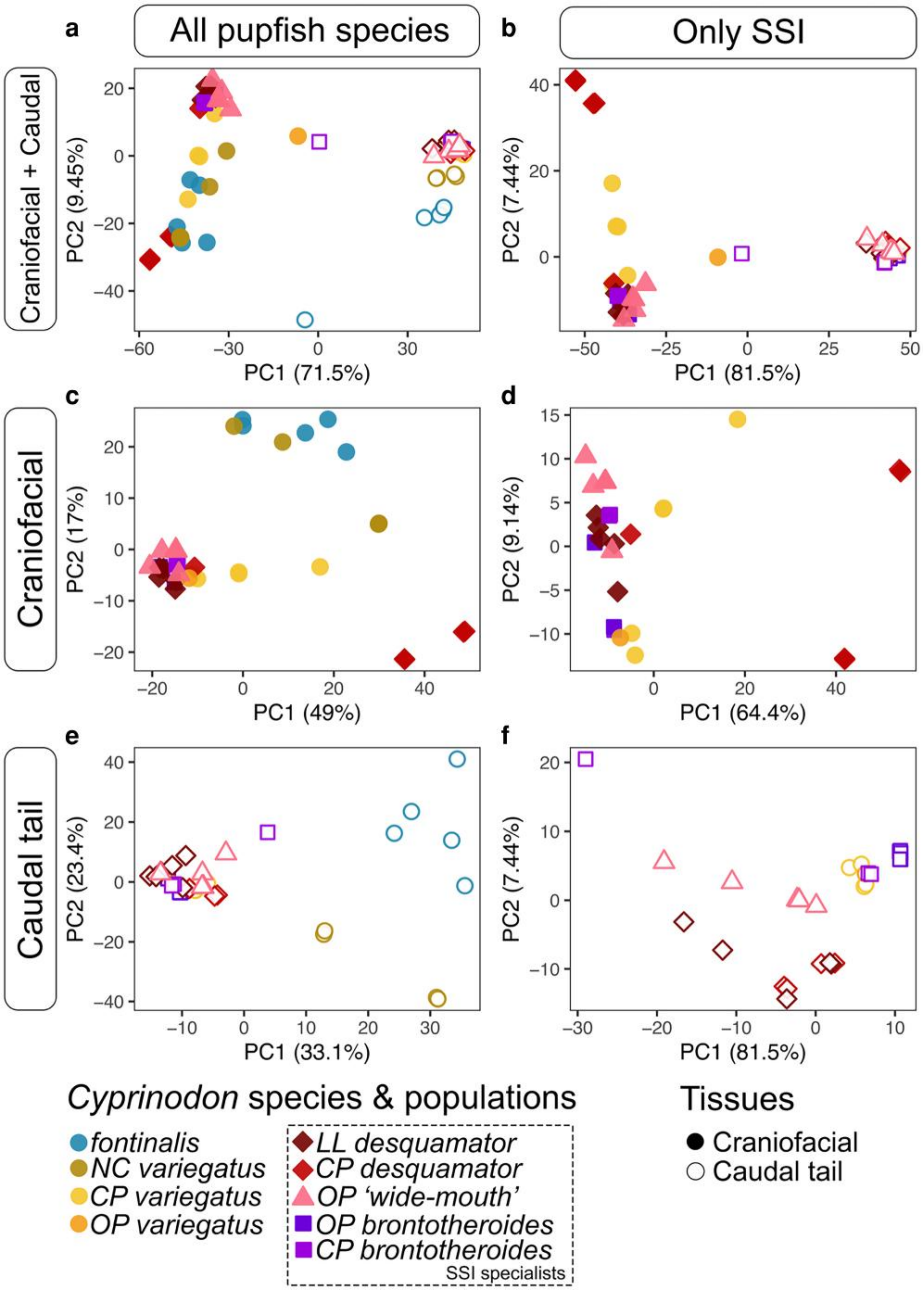
Principal component analyses of craniofacial and caudal-specific transcriptional variation across pupfish species. Each dot represents a sequenced RNA sample composed of either the dissected craniofacial (filled) or caudal regions (unfilled) pooled together from 5 to 6 hatched larvae at 8 d post-fertilization. a, b) combined craniofacial and caudal transcriptome samples across species; c, d) only craniofacial transcriptomes; and e, f) only caudal transcriptomes. First column: all 5 pupfish species sampled. Second column: SSI only (*C. variegatus*, *C. desquamator*, *C. brontotheroides*, and *C.* sp. “wide-mouth”). NC, North Carolina; SSI lakes: CP, Crescent Pond; OP, Osprey Lake; LL, Little Lake (see Fig. 1c for details).

Principal component analyses (PCA) across 4.3 billion mapped reads (Supplementary Fig. 1) revealed tissue type and species identity are the primary drivers of transcriptional divergences, explaining 71.5% and 9.5% of the variance, respectively (Fig. 2a). When restricted to SSI species only, variance explained by PC1 (primarily tissue type) increased to 81.5% (Fig. 2b). When pooled together by tissue, transcriptomes clustered primarily by species. PC1 explained 49% of the variance across all pupfish species, and 64.4% when restricted only to SSI species (Fig. 2c and d). For the caudal region, PC1 accounted for 33.1% of the variance across species and 81.5% among SSI species alone (Fig. 2e).

Notably, the craniofacial region of scale-eaters from Crescent Pond formed a distinct cluster from all other pupfish species, including a second lab-reared *desquamator* population originating from Little Lake (Fig. 2e and f, dark red) and from the intermediate scale-eater species, *Cyprinodon* sp. “wide-mouth” (Fig. 2e and f, pink) from Osprey Pond. This pattern of transcriptomic variance between the 2 *C. desquamator* populations within SSI is consistent with previous analyses showing clear genetic differentiation between these sister species (Richards and Martin 2017; McGirr and Martin 2021).

The elevated transcriptional variance observed between tissue transcriptomes, and among craniofacial transcriptomes in SSI specialists compared to generalist pupfishes, suggests ongoing regulatory divergence of developmental gene pathways among species during embryonic development, detectable at hatching. Collectively, these patterns of transcriptional divergence mirror the underlying genetic diversity observed in pupfishes in the Caribbean (Richards et al. 2021; Patton et al. 2022) and the pronounced adult craniofacial morphologies that make this young adaptive radiation so remarkable (Martin and Wainwright 2011).

### High proportions of DEGs unique to the craniofacial region in trophic specialists relative to the caudal region

To identify the genes contributing to the overall high transcriptional variance between specialist and non-specialist pupfishes, we employed DESeq2 (Love et al. 2014) to analyze differential gene expression between craniofacial and caudal tissues across pooled species comparisons. Our model included fixed independent variables for species (*n* = 6 when accounting for the North Carolina *C. variegatus ovinus*), population (*n* = 5; outside SSI: Chihuahua, North Carolina; within SSI: Crescent Pond, Little Lake, Osprey Lake), and dissected region (craniofacial and caudal). To detect genes exclusively differentially expressed for each species, we pooled all craniofacial or caudal transcriptome replicates from each given pupfish species (*C. fontinalis*, NC *C. variegatus*, SSI *C. variegatus*, *C. brontotheroides*, *C. desquamator*, and *Cyprinodon* sp. “wide-mouth”) across multiple populations and compared them pairwise against all other pupfish species’ corresponding craniofacial or caudal transcriptomes (Fig. 2a).

**Fig. 3.**
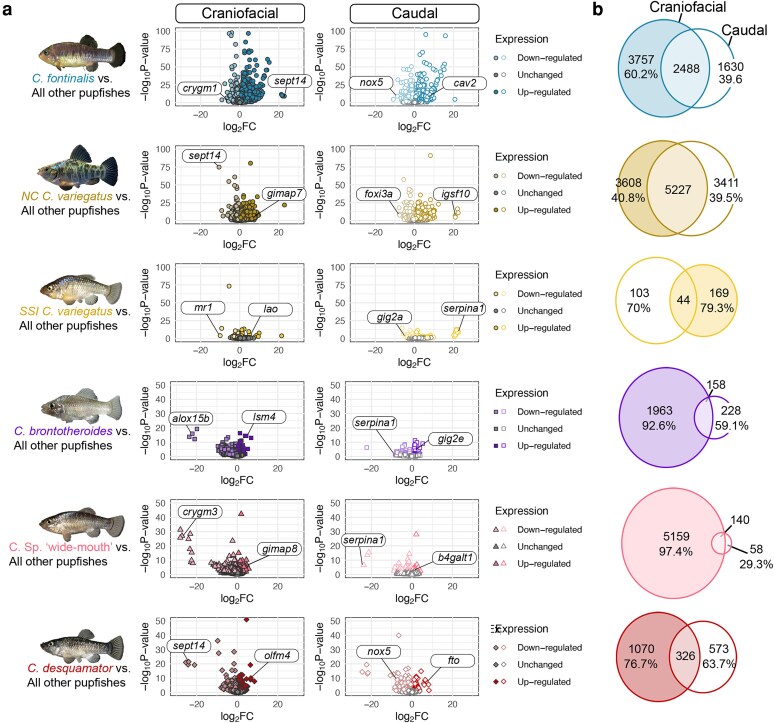
Differential gene expression in each species for each tissue type. Each row represents differentially expressed genes (DEG) unique to each species relative to all the other pupfish species in the dataset. SSI lake populations were pooled together by species in each comparison. a) Volcano plots show up- and down-regulated genes between species for each tissue region. The gene with the highest fold-change for craniofacial- and caudal-exclusive DEGs is annotated on each panel. b) The number of craniofacial and caudal DEGs for each comparison. Common DEGs between craniofacial and caudal tissues are represented as the intersection of left (filled) and right (unfilled) Venn diagrams. The percentages of exclusive craniofacial or caudal tissue DEGs relative to the total number of DEGs for each tissue are shown.

Our differential gene expression analysis revealed that the craniofacial region of SSI pupfishes exhibits a higher number of unique DEGs compared to the caudal region (Fig. 3b). Both the molluscivore *C. brontotheroide*s and the intermediate scale-eater *Cyprinodon* sp. “wide-mouth” showed the highest proportion of craniofacial-exclusive DEGs, with 92.6% and 97.4%, respectively (Fig. 3b: fourth and fifth row, purple and pink, respectively). This corresponded to 1,963 craniofacial-exclusive DEGs in the molluscivore (Fig. 3b: purple; see Supplementary File 1 for the gene list) and 5,159 craniofacial-exclusive DEGs in the “wide-mouth” (Fig. 3b: pink; see Supplementary File 1 for the gene list). Fewer than 10% of DEGs overlapped between craniofacial and caudal tissues in both species (8% and 2.7%, respectively), likely reflecting substantial transcriptional divergence in the craniofacial but not the caudal region, consistent with their highly specialized dietary niches.

Across generalist populations, in contrast, relatively fewer craniofacial-exclusive DEGs were found. For example, in the outgroup generalist *C. fontinalis*, 60.2% of the craniofacial DEGs were exclusive to the craniofacial tissue, and 42.8% of craniofacial DEGs in the North Carolina generalist were craniofacial-exclusive (Fig. 3b: first and second row, blue and mustard brown, respectively). This is consistent with the highly similar craniofacial morphology shared by these generalists, despite 30 to 100,000 years of divergence from SSI pupfishes. The *C. variegatus* population endemic to SSI however showed an increased proportion of craniofacial-exclusive DEGs, yet with the lowest number overall, with 444 craniofacial-exclusive and 141 caudal-exclusive DEGs (Fig. 3b: third row, yellow), over 6-fold reduction in DEGs compared to generalists from NC and Chihuahua.

Unexpectedly, in *C. desquamator*, the scale-eater pupfish with the most pronounced craniofacial differences due to its enlarged oral jaws, we found 76.7% of craniofacial-exclusive DEGs relative to all other pupfishes (Fig. 3b: sixth row), a proportion similar to craniofacial tissues in SSI generalists. To determine whether this lower percentage of craniofacial-exclusive transcripts in the scale-eaters reflects the higher transcriptional variance observed between lake populations within SSI (Fig. 2b, d, and e), we did a pairwise comparison between each of the *C. desquamator* populations and all the other pupfish species in our dataset (Supplementary Fig. 2). We indeed observed a higher percentage of craniofacial-exclusive DEGs in both *desquamator* populations separately. In the craniofacial tissues of Crescent Pond *desquamator* at hatching, 88.7% of craniofacial DEGs were craniofacial-exclusive (Supplementary Fig. 2, bright red), while in *desquamator* from Little Lake, 91.2% of craniofacial DEGs were exclusive (Supplementary Fig. 2, dark red). This variation in differential gene expression between *desquamator* populations is consistent with the genetic divergence previously reported between these 2 SSI populations (Turner et al. 2008; McGirr and Martin 2017; Richards et al. 2021; Patton et al. 2022), despite their nearly indistinguishable craniofacial morphologies (Martin 2016). We subsequently analyzed only pooled lake populations for *desquamator* similar to other SSI species.

Interestingly, in both populations of scale-eaters, the most downregulated gene in craniofacial tissues was *sept14*, while in the caudal region, *serpina1* and *fto* were the highest down- and up-regulated genes, respectively. *Serpina1* and *fto* remained the top differentially expressed craniofacial- and caudal-exclusive genes across both *desquamator* populations, suggesting parallel gene expression signatures.

We observed consistent differences in the number of craniofacial- vs caudal-exclusive DEGs across specialist pairwise comparisons (Fig. 3a, Supplementary Table 4). All specialists’ craniofacial tissues exhibited a greater number of exclusive DEGs than caudal tissues, highlighting a pervasive trend toward transcriptional regulation of genes putatively involved in derived craniofacial development.

### Genes with extreme fold-change reveal shared and tissue-specific expression shifts

Several genes identified as differentially expressed using DESeq2 showed extreme expression divergence, with absolute log_2_ fold changes exceeding ±20 (Fig. 3; Supplementary Table 5), which corresponds to more than a million-fold difference in normalized expression levels between species. The craniofacial-exclusive highest fold-change DEGs were unique for each species comparison, yet 2 caudal-exclusive DEGs (*nox5* and *serpina1*) were shared between 2 species with the same direction of expression. We also found that *alox15b* and *igsf10* showed the highest fold-change shared between tissues in *C. fontinalis*, and in the scale-eater *C. desquamator*, whereas *nox5* was shared between the 2 species in only caudal tissues (Fig. 3; Supplementary Table 5). The conservation of such extreme expression shifts across 2 morphologically and functionally distinct regions for *alox15b* and *igfs10* suggests a more general systemic role in the SSI radiation.

### Enriched gene ontology terms reveal divergent functional pathways between shared and unique craniofacial DEGs in trophic specialists

**Fig. 4.**
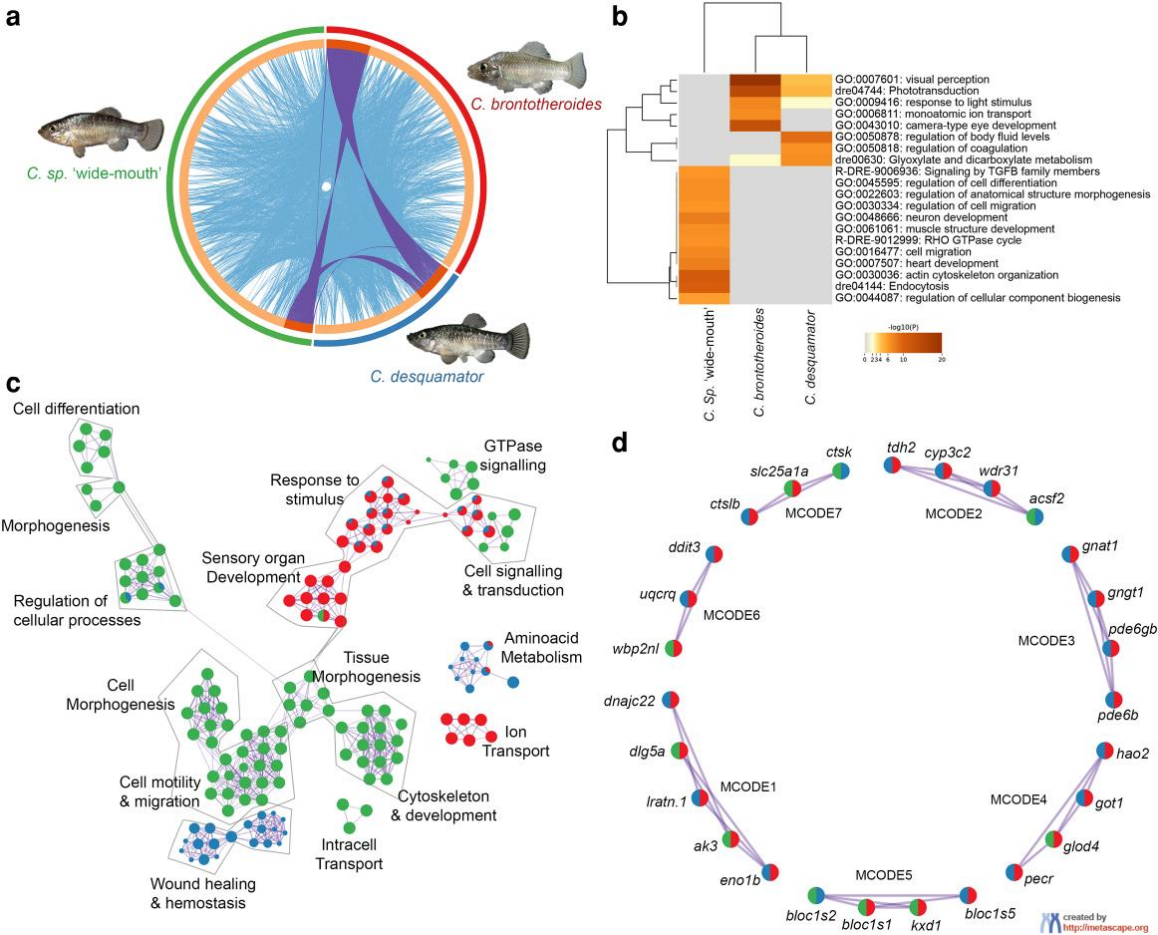
Functional enrichment and network visualization of craniofacial-exclusive DEGs in San Salvador Island trophic specialists. Gene ontology (GO) enrichment terms were identified using Metascape (Zhou et al. 2019). a) Circos plot showing shared and species-specific craniofacial-exclusive DEGs among *C. brontotheroides* (red), *C. desquamator* (blue), and *C.* sp. “wide-mouth” (green). Inner purple ribbons link identical genes between species, and blue ribbons link genes that belong to the same enriched ontology term. The inner circle represents the input multispecies lists (*n* = 1,963 for *C. brontotheroides*, *n* = 1,070 for *C. desquamator*, and *n* = 3,000 for *Cyprinodon* sp. “wide-mouth” the limit per sublist). Genes that hit multiple species lists are colored in dark orange, and genes unique to a single species list are shown in light orange. b) Heatmap of top enriched GO terms for each specialist. c) Enrichment network visualization of GO terms from all specialist-specific craniofacial-exclusive DEGs. Nodes represent GO terms. Node pie charts show gene list contributions by species. Labels were added manually. d) Representative MCODE protein–protein interaction subnetworks derived from the merged gene lists (*n* = 6,033). Each node represents a gene, and pie chart sectors denote species-level specificity. MCODE clusters highlight shared and species-specific modules, with MCODE3 (phototransduction) and MCODE5 (vesicle-mediated transport) having significant log_10_P values. Full enrichment results and complete network visualizations are available in Supplementary Figs. 5 to 7.

To identify biological processes and molecular pathways enriched in craniofacial-exclusive DEGs unique to trophic specialists (Fig. 4), we performed gene ontology (GO) analysis on shared and unique craniofacial DEGs using ShinyGo (Ge et al. 2020), PANTHER (Mi et al. 2017), and Metascape (Zhou et al. 2019). While all 3 methods produced broadly consistent enrichment results, we chose to visualize results using Metascape (www.metascape.org; Zhou et al. 2019) due to its integrated functional annotation, interactive network clustering, and high-quality graphical outputs. Overall, we found more shared DEGs overlapping between *C. brontotheroides* and *C. desquamato*r than with *C.* sp. “wide-mouth” (Fig. 4a, purple ribbons and dark orange versus light orange; see Supplementary File 1 for gene list). The top 3 most-enriched GO terms in the molluscivore craniofacial region were visual perception, phototransduction, and eye development, while regulation of body fluids, coagulation, and xylate metabolism were enriched in the *desquamator* craniofacial region (Fig. 4b). Craniofacial-exclusive DEGs in the intermediate scale-eater were enriched for endocytosis, actin cytoskeleton organization, and neuron development (Fig. 4b).

Network interactions of shared craniofacial-exclusive genes revealed functional clusters associated with sensory organ development, response to stimulus, cell signaling, and transduction (Fig. 4c). Amino acid metabolism, wound healing, and hemostasis were enriched in *desquamator* scale-eaters and they also shared enriched terms in regulation for cellular processes with the intermediate scale-eater, *C*. sp. “wide-mouth” (Fig. 4c). Similarly, Molecular Complex Detection (MCODE) (Bader and Hogue 2003) based protein–protein interaction network analysis identified enrichment for gene modules involved in phototransduction and vesicle-mediated transport between species (Fig. 4d, MCODE3 and MCODE5, respectively). To refine the analysis, we also examined the subset of craniofacial-exclusive DEGs shared by all 3 specialists (*n* = 61; Supplementary Fig. 4), which also revealed a striking enrichment for genes involved in phototransduction and visual perception (eg *gnat1*, *gngt1*, *pde6b*, and *pde6gb*), also supported by functional clustering and a shared MCODE module (MCODE1; Supplementary Fig. 5c, Supplementary File 2).

### Intersection of adaptive alleles with species-specific craniofacial DEGs reveals novel craniofacial gene candidates

Previous genomic scans of 202 Caribbean pupfish genomes identified 1,490 candidate adaptive single-nucleotide polymorphisms (SNPs) in *C. brontotheroides* and 3,463 in *C. desquamator*. These SNPs were characterized by (i) high genetic differentiation between specialist and generalist (Fst > 0.95) and (ii) located within genomic regions showing strong evidence of a hard selective sweep supported by linkage disequilibrium and site frequency spectrum-based statistics (Richards et al. 2021). Notably, most of the specialist candidate alleles near genes were found within the upstream 20 kb regulatory region rather than in coding or intronic regions (Richards et al. 2021), implicating a high potential for a regulatory role for these alleles in controlling gene expression. We also incorporated 51 candidate adaptive alleles from the newly discovered intermediate scale-eater, *C*. sp. “wide-mouth,” discovered using a similar pipeline (Richards and Martin 2022). Of the 1,490 candidate adaptive alleles found in *C. brontotheroides*, 578 mapped to 58 annotated genes; 1,528 of the 3,463 candidate adaptive alleles found in *C. desquamator* mapped to 168 genes; and 12 out of 51 candidate adaptive alleles in *Cyprinodon* sp. “wide-mouth” mapped to 7 genes. We hypothesized that intersecting our specialist-specific craniofacial-exclusive DEGs with this list of adaptive alleles would reveal promising genes in which genetic regulatory divergence contributes to the observed craniofacial-exclusive gene expression differences found at hatching in each of the specialist lineages.

**Fig. 5.**
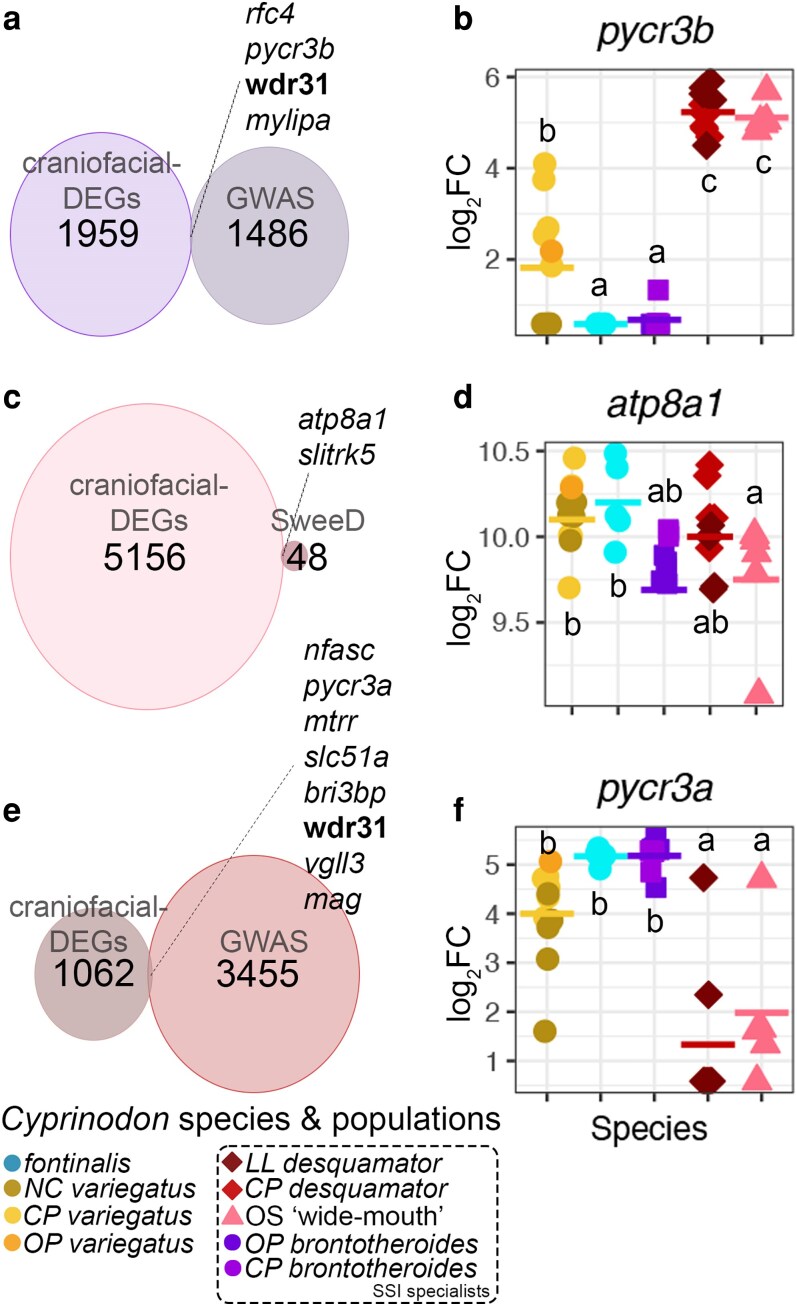
Overlap between species-specific craniofacial DEGs and candidate adaptive alleles in each trophic specialist (below). Intersection between species-specific craniofacial DEGs (left circles; from Fig. 3) and candidate adaptive alleles (right circles; identified from genome-wide scans for highly differentiated SNPs within hard selective sweeps (Richards et al. 2021; Richards and Martin 2022) in a) *C. brontotheroides*, c) *Cyprinodon* sp. “wide-mouth,” and e) *C. desquamator*. Circles are scaled by the number of craniofacial DEGs they contain. Dashed lines indicate the set of overlapping genes for each specialist. Counts across species and populations for the top intersected genes are shown in d to g. *Wdr31* (bolded) is a craniofacial-exclusive DEG with adaptive alleles in both *brontotheroides* and *desquamator*. b, d, f) Compact letter display based on Tukey-adjusted HSD post hoc test pairwise comparisons (different letters signify *P* < 0.05).

This intersection of specialist adaptive alleles (Richards et al. 2021; Richards and Martin 2022) and specialist-specific craniofacial-exclusive DEGs (this study) pointed to only a few candidate genes (14 in total across species), all of which lack known craniofacial expression or function. We identified *rfc4*, *pycr3b*, *wdr31*, and *mylipa* to be the only *brontotheroides*-specific craniofacial-exclusive DEGs that overlapped with candidate adaptive alleles in the molluscivore population (Fig. 5a). Two genes, *atp8a1* and *slitrk5*, as *C*. sp. “wide-mouth”-specific craniofacial DEGs which overlapped with candidate adaptive alleles in this population (Fig. 5c). And for the *desquamator* scale-eater, we found *pycr3a*, *slc51a*, *bri3bp*, *vgll3*, *nfasc*, *mtrr*, and *mag* as the only craniofacial-exclusive DEGs overlapping scale-eater adaptive alleles in this species (Fig. 5e). Interestingly, both paralogs of *pycr3*, *pycr3a* and *pycr3b*, showed species-specific differential gene expression in SSI specialists, and *wdr31* was the only gene shared by both *brontotheroides* and *desquamator*. To explore variation in gene expression of these novel craniofacial candidate genes, we quantified their mean normalized log_2_-transformed read counts across samples within and outside SSI (Fig. 5b, d, and f; Supplementary Fig. 3).

### Divergent expression of *pycr3* paralogs in specialist craniofacial tissues correlates with overall expression differences and multiple shared SNPs within the *pycr3* locus

Members of the pyrroline-5-carboxylate reductase family, including *pycr3*, catalyze the final steps in the biosynthesis of proline with known roles in cancer progression (Burke et al. 2020; Bogner et al. 2021; Zhuang et al. 2024) but an unknown role in craniofacial development.

We observed divergent expression for the paralogs *pycr3a* (position KL653062.1: 39,029–40,716) and *pycr3b* (position KL653062.1: 32,932–34,621) in 2 of the 3 specialists. *Pycr3a* was downregulated and *pycr3b* upregulated in both scale-eaters, *C.* sp. “wide-mouth” (Fig. 5b) and *C. desquamator* (Fig. 5f), compared to all other pupfishes. *Pycr3a* and *pycr3b* share 99% nucleotide sequence identity, making it nearly impossible to distinguish their spatial expression. Neither paralog was expressed in the caudal tissues of any pupfish species.

**Fig. 6.**
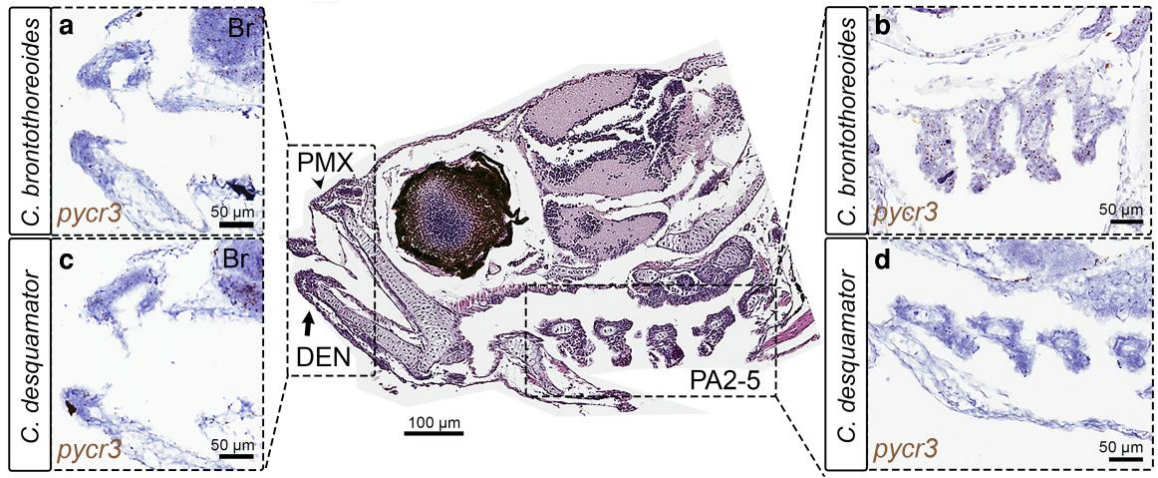
*Pycr3* expression is observed in the pharyngeal arches of molluscivores but absent in scale-eaters at hatching. RNAscope 2.5 ISH for *pycr3* (note: dark brown spots indicate probe binding) at hatching (8 dpf): a, b) *C. brontotheroides*; c, d) *C. desquamator*. Note the absence of expression in scale-eater pharyngeal arches (d). Center panel shows hematoxylin and eosin (H&E) stained sagittal section of a Little Lake *C. desquamator* hatchling, highlighting craniofacial structures including the second to fifth pharyngeal arches (PA2-5), premaxilla (PMX, arrowhead), and dentary (DEN, arrow).

To investigate the potential differential expression of *pycr3* between craniofacial tissues of the specialists, we used RNAscope in situ mRNA hybridization (ISH) assays at hatching (8 dpf) with automated sectioning and staining performed by the Pathology Services Core Facility at the University of North Carolina at Chapel Hill. Consistent with the low craniofacial expression of *pycr3a* observed in scale-eater and “wide-mouth” tissues (Fig. 5f), we observed the absence of *pycr3* expression in situ in the posterior skeletal elements of the jaw (pharyngeal arches 2 to 5) in *C. desquamator* (Fig. 6d, *n* = 2). However, in *C. brontotheroides*, *pycr3* was abundantly expressed across pharyngeal arches 2 through 5 (Fig. 6b, *n* = 2). We also found consistent expression of *pycr3* in the premaxilla and future dentary bone of both molluscivores and scale-eaters, as well as in the brain (Fig. 6a and c, *n* = 4).

### Divergent expression of *atp8a1* in craniofacial tissues between the scale-eater and generalist

**Fig. 7.**
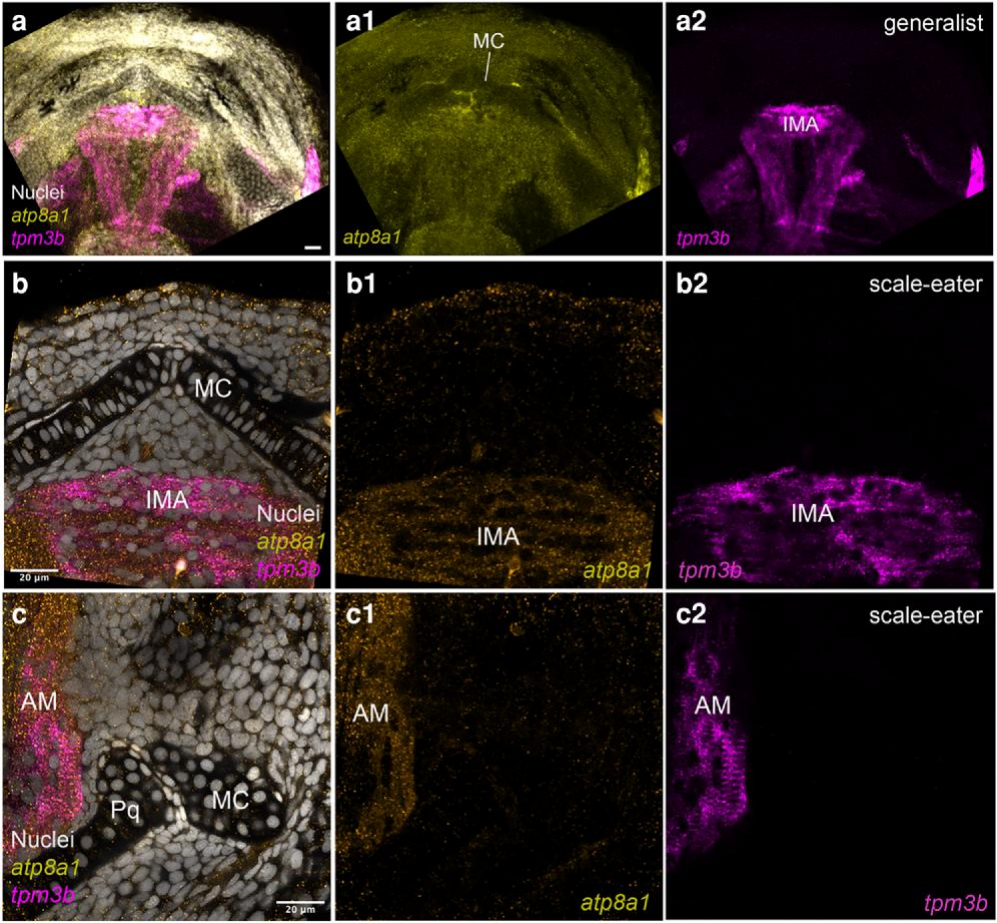
*Atp8a1* expression is restricted to the developing intermandibular anterior and adductor mandibulae craniofacial muscles in scale-eaters. Single optical section of fluorescent HCR *atp8a1* (orange) and *tpm3b* (magenta) in the lower jaw of a) generalist and b, c) scale-eater at hatching (8 dpf). a1 to c1) Single channel for *atp8a1*. a2 to c2) Single channel for *tpm3b*. MC, Meckel's cartilage; IMA, intermandibular anterior muscle; AM, adductor mandibulae muscle. Nuclei labeled with DAPI.

With previously unknown craniofacial function but ubiquitous expression in humans, *atp8a1* (ATPase phospholipid transporting 8A1) catalyzes ATP hydrolysis coupled with aminophospholipid transportation controlling phospholipid asymmetry across the membrane (Hiraizumi et al. 2019). To investigate the spatial differential expression of *atp8a1* in craniofacial tissues between species, we performed fluorescent hybridization chain reaction (HCR) for *atp8a1* and *tpm3b* (*tropomyosin 3b*), a marker for cranial muscle (Palominos et al. 2023). *Atp8a1* was broadly expressed in craniofacial tissues of generalists (Fig. 7a to a2) at hatching, whereas in scale-eaters its expression was mainly restricted to the intermandibular anterior (IMA) and the adductor mandibulae (AM) muscles, corroborated by the colocalization with *tpm3b* (Fig. 7b and c).

### Increased craniofacial transcriptional variance is associated with exceptional craniofacial diversification rates

We observed substantially higher transcriptional variance in craniofacial tissues (Fig. 2c, PC1: 48.6%) than in caudal tissues (Fig. 2e, PC1: 33.1%), a pattern that was consistent even when limited to San Salvador Island species (PC1: 61.3% vs. PC1: 22.9% in craniofacial versus caudal tissues, respectively). This elevated variance is associated with exceptionally fast rates of morphological diversification observed primarily in craniofacial traits, while the caudal region has remained relatively conserved across *Cyprinodon* species (Martin and Wainwright 2011; Martin 2016). Interestingly, the largest effect sizes for the 30 trait-associated QTL examined to date have also been identified only in craniofacial traits, accounting for up to 15% of oral jaw size variation (Martin et al. 2017). Our findings suggest that rapid morphological diversification may be linked to elevated transcriptional variance at hatching.

Furthermore, the elevated transcriptional variance observed in craniofacial tissues between pupfish species is reflected at the genetic regulatory level: specialists have an increased number of highly differentiated SNPs under selection within upstream gene regulatory regions compared to generalists (McGirr and Martin 2017; Richards et al. 2021). This suggests that the increased expression variance may result from different cis-regulatory variants between specialist species, contributing to stable, heritable shifts in gene expression between species that are observable at hatching time.

The increased transcriptional variance observed in craniofacial tissues thus aligns with the idea that large-scale morphological changes often arise through mutations in regulatory sequences (King and Wilson 1975; Prud’homme et al. 2007) reflecting genetically encoded species-specific developmental shifts that facilitate craniofacial morphological evolution in each lineage.

### Craniofacial-specific transcriptional outliers highlight shared modes of gene expression divergence

Our analyses revealed that specialist pupfishes exhibit a strikingly high proportion of craniofacial-exclusive DEGs compared to generalists outside SSI. Both the molluscivore (*C. brontotheroides*) and the intermediate scale-eater (*Cyprinodon* sp. “wide-mouth”) displayed over 90% of DEGs unique to craniofacial tissues. This tissue specificity contrasts with patterns observed in generalist species, in which a substantial portion of DEGs were shared between craniofacial and caudal tissues. These findings suggest that regulatory changes underlying dietary specialization in these species disproportionately target craniofacial gene networks, without widespread transcriptional changes in slower-evolving tissues such as the caudal region.

Although the precise mechanisms remain unknown, this disproportionate enrichment of craniofacial-exclusive DEGs in specialists may enable fine-tuned morphological innovations in the head that are essential for specialist feeding strategies while preserving ancestral (generalist) body plans elsewhere. This pattern is consistent with models of regulatory evolution in which spatially restricted cis-regulatory changes allow for morphological innovation with minimal pleiotropic effects (Prud’homme et al. 2007; Cleves et al. 2014; Mack et al. 2022).

At the same time, we also identified a small set of transcriptional outliers—*alox15b*, *sept14*, and *igsf10*—that were among the most highly differentially expressed genes across both craniofacial and caudal tissues in multiple pupfish species (Supplementary Table 5) with over a million-fold difference in normalized expression. Notably, between the distant outgroup *C. fontinalis* and the scale-eater *C. desquamator*, *alox15b* (*arachidonate 15-lipoxygenase type B*) and *igsf10* (*immunoglobulin superfamily member 10*) were differentially expressed in both craniofacial and caudal tissues suggesting that some regulatory changes may be under strong directional selection despite their more central roles in broader metabolic and physiological processes.

*Sept14* (*septin 14*), a member of the GTP-binding septin family of cytoskeletal proteins involved in cell division and host defense (Kinoshita 2008; Mostowy and Cossart 2012), emerged as the top craniofacial-exclusive DEG in *C. fontinalis* (upregulated) and the scale-eater *C. desquamator* (downregulated) (Fig. 3a, Supplementary Table 5). Septin family genes in zebrafish are broadly expressed throughout the body and play critical roles in immune defense against bacterial infection (Torraca et al. 2023) and apoptosis (Landsverk et al. 2010). The contrasting expression of *sept14* between the ancestral Chihuahuan pupfish and the scale-eater from SSI suggests an overall important role in craniofacial developmental divergence between these 2 species.

### Craniofacial expression of *pycr3* highlights a role for proline metabolism in the evolution of scale-eater craniofacial divergence

Unlike Pycr1 and Pycr2, which are found in mitochondria, Pycr3 localizes in the cytosol, catalyzing the synthesis of proline from ornithine-derived Δ1-pyrroline-5-carboxylate (P5C). Proline is a key amino acid used for collagen synthesis and extracellular matrix remodeling (Phang et al. 2015) and is also required for osteoblast differentiation and bone formation (Shen et al. 2022).

Even though in low abundance, our pipeline allowed us to correlate high *pycr3a* gene expression in the craniofacial transcriptomes of molluscivores (Fig. 5f) with in situ mRNA hybridization data corroborating the higher expression of a common *pycr3* RNA probe (due to the highly similar transcripts of *pycr3a* and *pycr3b*) in the pharyngeal arches of *C. brontotheroides* (Fig. 6b), and a lack of signal in the same structures in *C. desquamator* (Fig. 6d). This was independently supported by a recent zebrafish *pycr3* knockout model (Zeynalzadeh 2021) that revealed abnormalities in skeletal structures and CNC derivatives in embryos and larvae, including spinal curvature, abnormal jaw size, and a reduced head and eye size. Proline metabolism is unaffected in *pycr3^−/−^* zebrafish embryos suggesting that proline metabolism is compensated by other effectors such as Pycr1 or Pycr2.

To investigate whether other members of this gene family were differentially expressed and craniofacial-exclusive, we further analyzed the expression of the annotated *pycr1a* and *pycr1b g*ene products in our dataset (in the *C. variegatus* genome, there's no annotation for *pycr2*). We found that only *pycr1a* is differentially expressed in the craniofacial tissues of the outgroup generalist *C. fontinalis* and all the other pupfish, including the SSI generalist (Supplementary Fig. 8a). Unlike *pycr3a* and *pycr3b*, *pycr1a* and *pycr1b* are expressed in the caudal region and not differentially expressed between generalist and specialist species. This divergence in *pycr3* paralog expression, in contrast to the broader and more stable expression of *pycr1* paralogs across species and tissues (Supplementary Fig. 8), together with the role of proline metabolism on cartilage and bone development supports a model where *pycr3* expression changes contribute to divergent craniofacial development through cis- or trans-regulatory changes.

### Novel differential expression of atp8a1 supports a role for divergent muscle development in scale-eaters’ craniofacial development

Atp8a1 catalyzes the coupled hydrolysis of ATP and PS translocation to facilitate the formation of plasma membrane vesicles (Best et al. 2019; Hasegawa et al. 2021; Kook et al. 2021). Ubiquitously expressed, Atp8a1 localizes to all membrane-trafficking organelles (the trans-Golgi network, endoplasmic reticulum, endosomes, and lysosomes (Kook et al. 2021; Pocognoni et al. 2024)), and mutations in human Atp8a1 are linked to chronic and progressive liver diseases (Panatala et al. 2015). Knockout mice show no apparent morphological abnormalities apart from disrupted hematopoietic stem cell pool homeostasis (Zheng et al. 2023) and behavioral hyperactivity with impaired hippocampus-associated learning (Levano et al. 2012).

Prior to this work, *atp8a1* expression in fishes was uncharacterized. We found reduced expression of *atp8a1* in both SSI scale-eaters, *C. desquamator*, and *Cyprinodon* sp. “wide-mouth.” The restricted expression of *atp8a1* to *tpm3b*-positive craniofacial muscles, such as the AM and IMA, in *C. desquamator* scale-eaters (Fig. 7b and c) suggests that *atp8a1* expression is subject to different spatiotemporal regulation in scale-eaters than in generalists. Evidence of reduced chromatin accessibility in regulatory regions of *atp8a1* linked to high *atp8a1* transcript counts in atrophied muscles in mice (Lin et al. 2023), showing a responsive regulatory network activating *atp8a1* transcription. Our findings reveal a divergent pattern of *atp8a1* expression between the craniofacial muscles of scale-eaters and generalist pupfish at hatchling, suggesting the possibility of different roles in either or both craniofacial development and evolution.

It remains to be determined whether *pycr3* and *atp8a1* are expressed in neural crest cells or the pharyngeal mesoderm during early pupfish embryogenesis, respectively. Given their expression at hatching stage, we hypothesize that *pycr3* may be found expressed in cranial neural crest cells of generalists and molluscivores at the pharyngula stage, whereas *atp8a1* may be expressed in the pharyngeal mesoderm, as it later shows expression in the craniofacial muscles of pupfish hatchlings (this study) and in both developing and adult mammalian skeletal muscle [from Gene Expression Omnibus (GEO), Bethesda (MD): National Center for Biotechnology Information (USA); 1999].

## Conclusion

Understanding how genetic variants regulate gene expression and drive morphological variation among species is fundamental for understanding the genetic basis of both human diseases and adaptive evolution. In this study, we used tissue-specific bulk RNA sequencing within a nascent adaptive radiation of pupfishes endemic to San Salvador Island to investigate the genetic changes underlying differential gene expression during development among highly morphologically divergent trophic specialists. From over 5 million segregating single-nucleotide polymorphisms and thousands of loci previously associated with oral jaw divergence in these species (Richards et al. 2021; Richards and Martin 2022; McGirr and Martin 2021), we pinpointed a small subset (*n* = 14) that overlap with craniofacial-exclusive differentially expressed genes (DEGs) unique to craniofacial tissues in pupfish trophic specialists at a single timepoint (hatching: 8 dpf).

We additionally validated divergent and novel craniofacial expression in 2 of these genes using 2 methods *for* in situ mRNA hybridization (RNAscope and HCR), focusing on the 2 most prominent candidates in this study. Notably, all 3 candidate genes investigated thus far, including *galr2* from a previous study (Palominos et al. 2023), have revealed novel expression patterns in the craniofacial tissues of pupfishes that have not been previously associated with craniofacial development in any vertebrate system. These results, representing an unexpected success rate of 100% (3 out of 3 candidates), suggest critical roles for these genes in controlling the development of divergent craniofacial morphologies in pupfishes. This demonstrates the power of the pupfish system to discover novel gene functions and sheds light on the regulatory mechanisms shaping craniofacial evolution. Moreover, our findings highlight the utility of integrating tissue-specific transcriptomics with evolutionary genomics to reveal new insights into the genetic architecture of adaptive morphological divergence.

## Supplementary Material

iyaf207_Supplementary_Data

## Data Availability

All sequencing reads generated by this study are deposited in the NCBI Sequence Read Archive as PRJNA1245446. Scripts for analysis and visualization of the results are publicly available at github.com/fishfena/JAWesome_RNAproject. Gene lists with craniofacial and caudal DEGs for each species comparison can be found here (Palominos et al. 2024): https://doi.org/10.5061/dryad.dncjsxm8c. Supplemental material available at GENETICS online.
