# Supplementary material for: Craniofacial-specific transcriptomics uncovers novel genes underlying jaw divergence in dietary specialist pupfishes
Source: Genetics. 2025 Sep 26;231(4):iyaf207. doi: 10.1093/genetics/iyaf207 (PMC12693500; doi:10.1093/genetics/iyaf207)
Supplement: iyaf207_Supplementary_Data [file iyaf207_supplementary_data.zip › Supplemental_Material_GENETICS-2025-308388.docx]

**Supplemental Materials**

**Craniofacial-specific transcriptomics uncovers novel genes underlying jaw divergence in dietary specialist pupfishes**

M. Fernanda Palominos^1,2^, Vanessa Muhl^1,2^, Christopher H. Martin^1,2^

^1^﻿Department of Integrative Biology, University of California, Berkeley, Berkeley, CA, 94720 U.S.A.

^2﻿^Museum of Vertebrate Zoology, University of California, Berkeley, Berkeley, CA, 94720, U.S.A.

Corresponding author

E-mail: [mfpalominos@berkeley.edu](mailto:mfpalominos@berkeley.edu) (MFP)

**Supplemental Figures:**

Figure S1- S8, Pages 1 - 7.

**Supplemental Tables:**

Tables S1-S5, Pages 7-9.

**Supplemental Files:**

Two .csv files (SupplementallFile1_CraniofacialDEGs.csv; SupplementalFile2_GOenrichment.csv) containing the list of differentially expressed genes (DEGs) in craniofacial for each specialist and the list of gene enriched for GO terminology.

**Short Title**

Craniofacial-specific gene expression underlying craniofacial evolution in pupfishes

**Supplemental Figures**

**
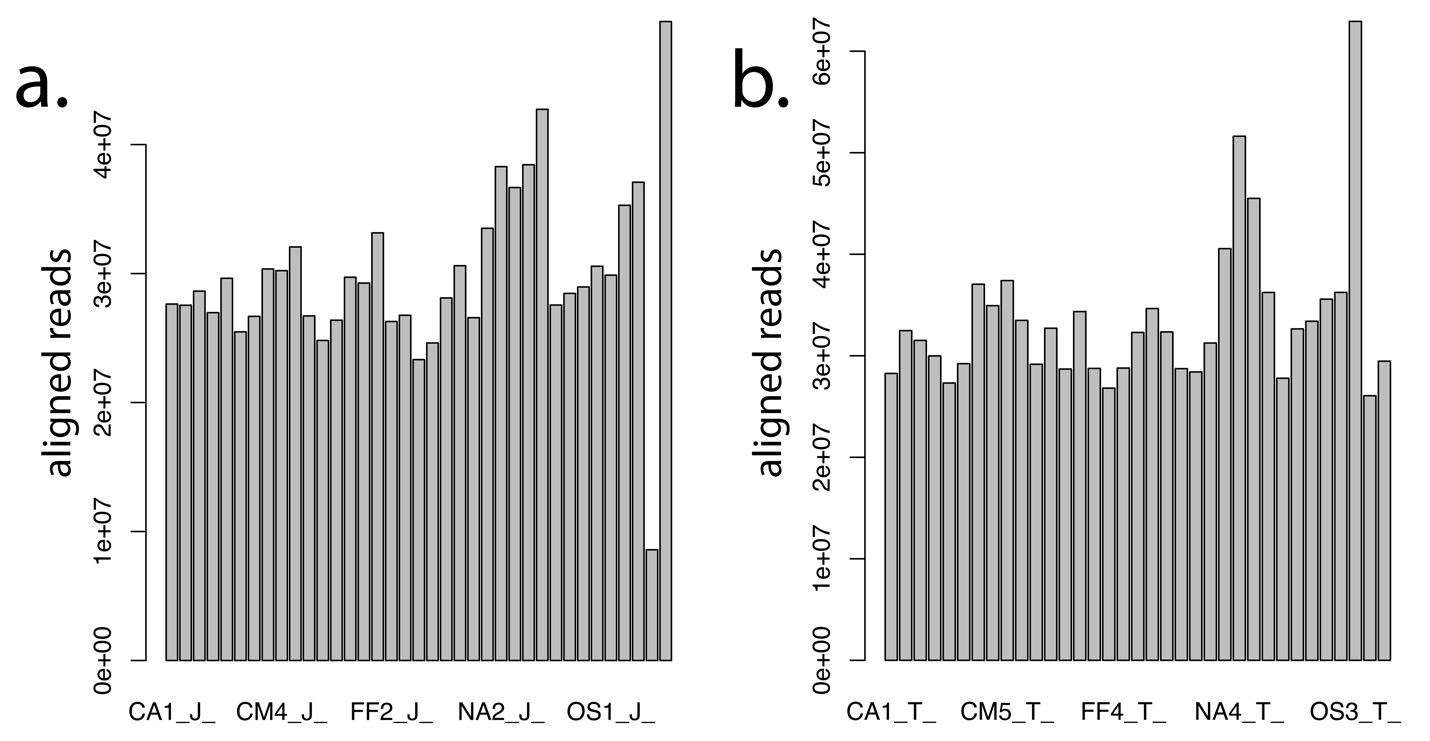
**

**Figure S1.** Number of aligned reads in alphabetical order for a) craniofacial and b) caudal tail sample tissues. CRP: Crescent Pond, OSP: Osprey Lake, NC: North Carolina, LIL: Little Lake; A: *variegatus*, M: *brontotheroides*, P: *desquamator*, FF: *fontinalis*.

**
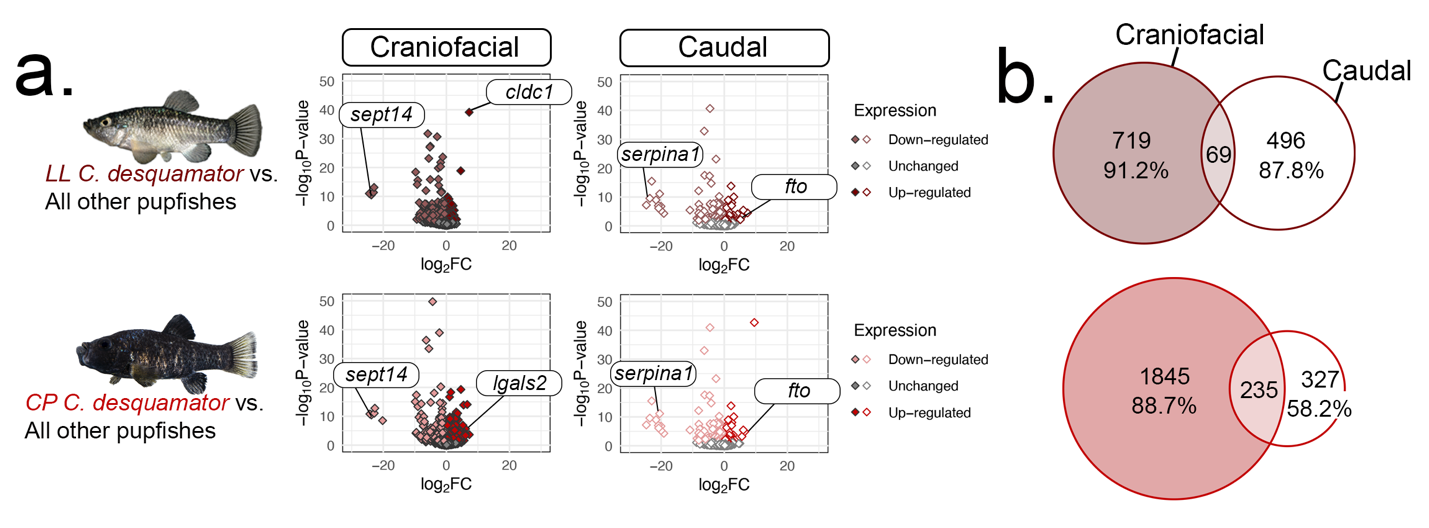
**

**Figure S2. Differential gene expression in two different lake populations of *C. desquamator* relative to all other pupfishes.** Craniofacial (filled symbols) and caudal region tissue (open symbols) for Little Lake (first row, dark red) and Crescent Pond (second row, light red). a) Volcano plots showing up- and downregulated genes between compared species, in each tissue. Filled symbols represent craniofacial samples (left panels) and open symbols represent caudal tissue dissections (right panel). For the craniofacial panels, the genes with the highest fold-change are annotated in the Volcano Plots. b) The number of craniofacial and caudal differentially expressed genes (DEGs) for each comparison. Common DEGs between craniofacial and caudal tissues are represented as the intersection of the left (filled) and right (unfilled) Venn diagrams. The percentages of exclusive craniofacial or caudal-tissue DEGs relative to the total DEGs for each tissue are shown.

**Figure S3 (next page). Expression across tissues and populations of the overlap between craniofacial-exclusive DEGs and adaptive alleles (main Figure 5).**


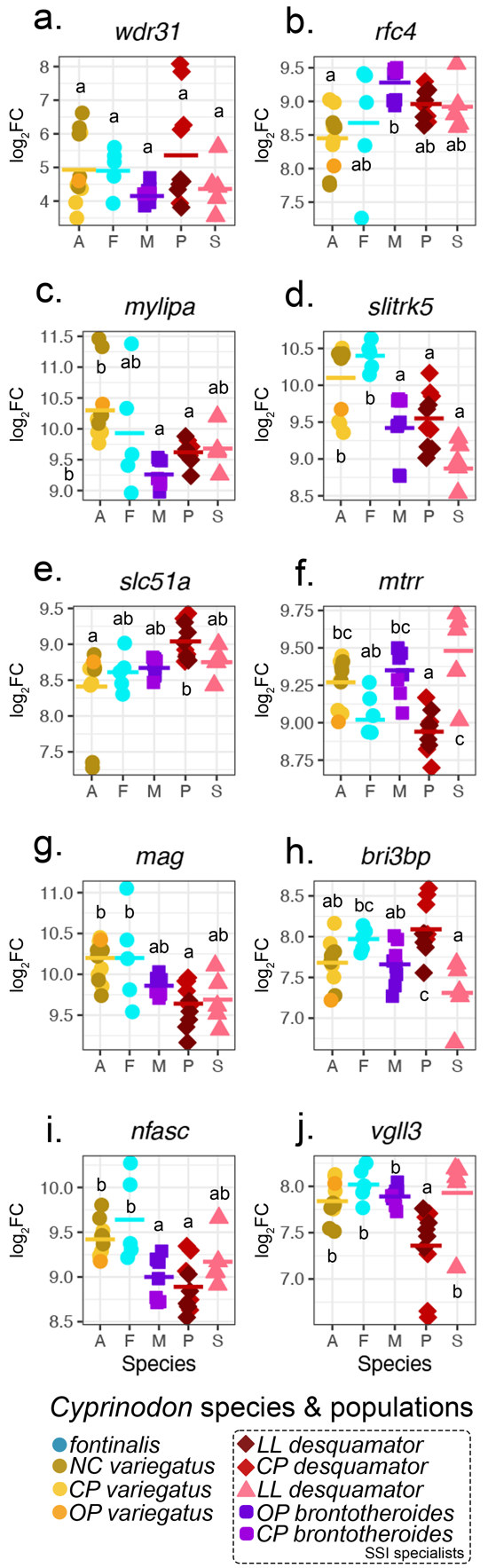


**
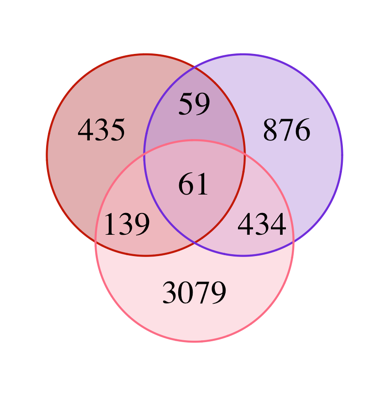
**

**Figure S4. Common and species-specific craniofacial-exclusive DEGs across *C. brontotheroides (purple, right), C. desquamator (brown, lwft)* and *C. sp*. ‘wide-mouth’ (pink, bottom).**

**
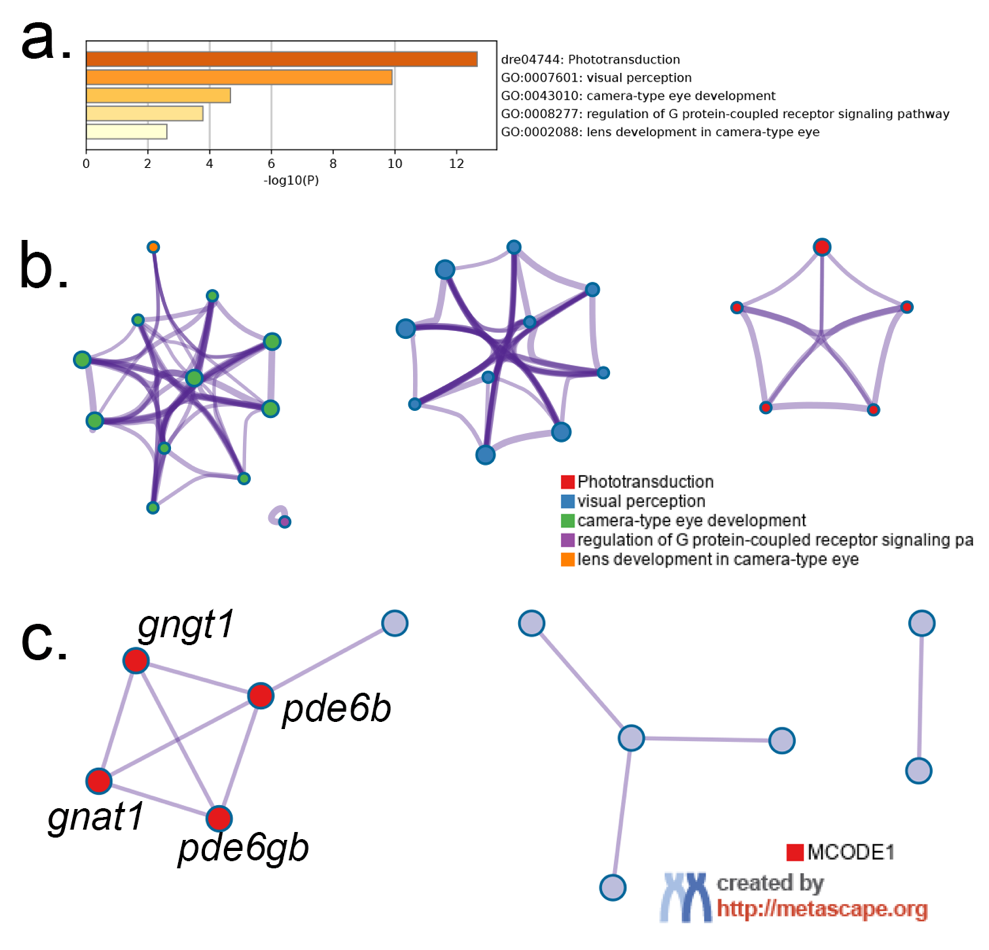
**

**Figure S5. Metascape full enrichment for common craniofacial-exclusive DEGs among trophic specialists.** a) Heatmap of top GO terms. b) Network of enriched terms colored by MCODE clusters. Nodes that share the same cluster ID are typically close to each other. c) MCODE1: Phototransduction. c) Protein-protein interaction network and MCODE components identified in the gene list. MCODE1: Phototransduction.

**
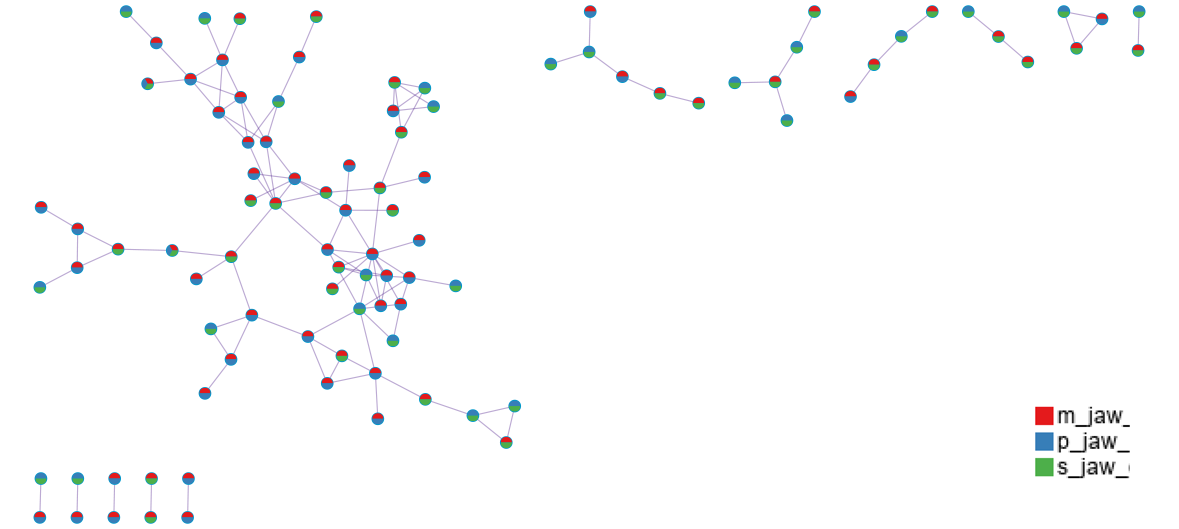
**

**Figure S6. Metascape full enrichment for multispecies craniofacial-exclusive across trophic specialists DEGs, colored by clust**

**
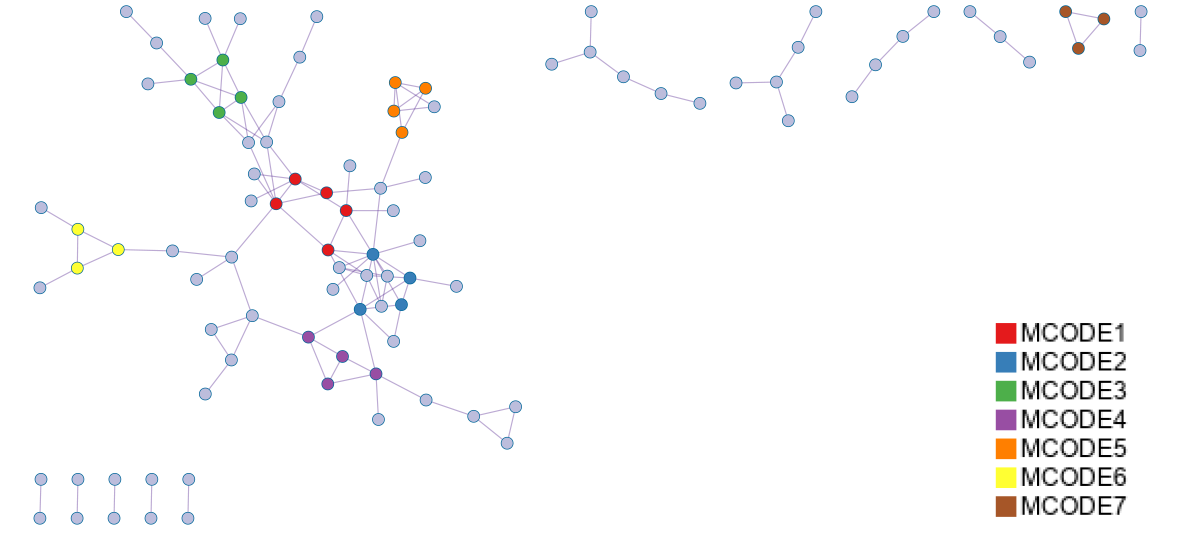
**

**Figure S7. Metascape full enrichment for multispecies craniofacial-exclusive across trophic specialists DEGs, colored by MCODE PPI clusters.**


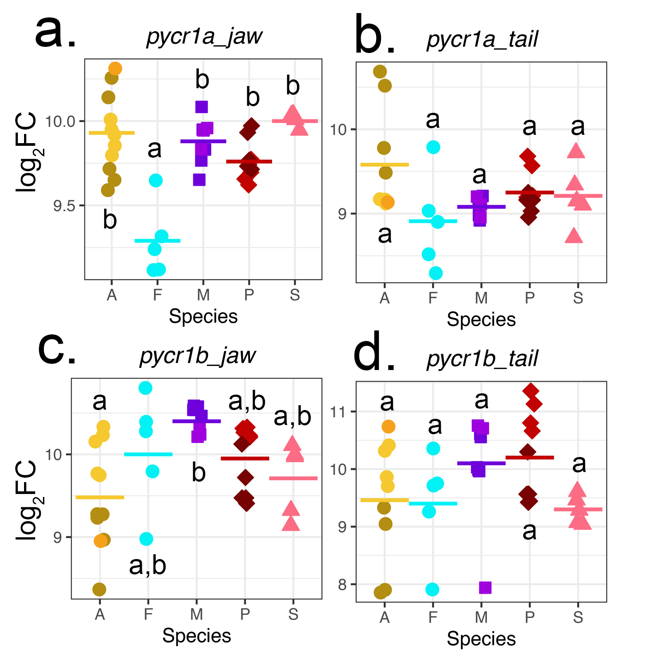


**Figure S8. *Pycr1a* and *pycr1b* expression across tissues and populations.**

**Supplemental Tables**

**Table S1.** Number of replicates for each sequenced transcriptomic sample. Populations are abbreviated as, CRP: Crescent Pond, OSP: Osprey Lake, NC: North Carolina, LIL: Little Lake.

| ***Cyprinodon*** | **Population** | **Tissue** | ***# Replicates*** |
| --- | --- | --- | --- |
| *variegatus* | CRP | Craniofacial | 5 |
| *variegatus* | OSP | Craniofacial | 1 |
| *variegatus* | NC | Craniofacial | 5 |
| *brontotheroides* | CRP | Craniofacial | 5 |
| *brontotheroides* | OSP | Craniofacial | 3 |
| *desquamator* | CRP | Craniofacial | 5 |
| *desquamator* | LIL | Craniofacial | 5 |
| *wide-mouth* | OSP | Craniofacial | 5 |
| *fontinalis* |  | Craniofacial | 5 |
| *variegatus* | CRP | Caudal | 4 |
| *variegatus* | OSP | Caudal | 1 |
| *brontotheroides* | CRP | Caudal | 4 |
| *brontotheroides* | OSP | Caudal | 5 |
| *desquamator* | CRP | Caudal | 3 |
| *desquamator* | LIL | Caudal | 4 |
| *wide-mouth* | OSP | Caudal | 5 |
| *fontinalis* |  | Caudal | 5 |

**Table S2.** Median number of reads and median percentage of alignment to *Cyprinodon* *variegatus* reference genome (C_variegatus-1.0, GCA_000732505.1) per species and tissue samples (the mean between all biological replicates). CRP: Crescent Pond, OSP: Osprey Lake, NC: North Carolina, LIL: Little Lake; A: *variegatus*, M: *brontotheroides*, P: *desquamator*, FF: *fontinalis*.

| **Population-Species-Tissues** | **# of input reads per specie** | **# of uniquely mapped reads per specie** | **Median % of alignment per species** |
| --- | --- | --- | --- |
| CRP-A-J | 27295152.5 | 17995054.5 | 65.9 |
| CRP-M-J | 25569728 | 17663993.5 | 69.1 |
| CRP-P-J | 26458976.5 | 17022724.5 | 64.3 |
| CRB-J | 27324647 | 17227715 | 63.0 |
| LIL-P-J | 28592652 | 18196382 | 63.6 |
| N-A-J | 27452332.5 | 17350981 | 63.2 |
| OSP-A-J | 27491209.5 | 17262840 | 62.8 |
| OSP-M-J | 27907245 | 18653858.5 | 66.8 |
| OSP-S-J | 21931034 | 14732769.5 | 67.2 |
| CRP-A-T | 27281556 | 18034858 | 66.1 |
| CRP-M-T | 27628940.5 | 18582541 | 67.3 |
| CRP-P-T | 28201746.5 | 18222015.5 | 64.6 |
| CRB-T | 28135282.5 | 17539540.5 | 62.3 |
| LIL-P-T | 29139598.5 | 18858055 | 64.7 |
| N-A-T | 28184310 | 16557850.5 | 58.7 |
| OSP-A-T | 32548716.5 | 21863651 | 67.2 |
| OSP-M-T | 28171658 | 18457117.5 | 65.5 |
| OSP-S-T | 34865607 | 22229652 | 63.8 |

**Table S3.** Total number of expressed genes per species and tissue samples (the mean between all biological replicates). A: *variegatus*, M: *brontotheroides*, P: *desquamator*, FF: *fontinalis*, N: North Carolina *C. variegatus*, C. sp. ‘wide-mouth’.

| **Species** | **Craniofacial** | **Caudal** |
| --- | --- | --- |
| A | 20380 | 18385 |
| M | 20389 | 18375 |
| P | 20389 | 18375 |
| FF | 20389 | 18375 |
| N | 20377 | 18462 |
| S | 20389 | 18378 |

**Table S4.** Craniofacial- and caudal-exclusive DEG across species comparisons with DESeq2.

| **Species comparison** | **Craniofacial-exclusive** | **Caudal-exclusive** | **Shared** |
| --- | --- | --- | --- |
| *A vs all other pupfishes* | 3757 | 1630 | 2488 |
| *NC vs all other pupfishes* | 3608 | 3411 | 5227 |
| *SSI A vs all other pupfishes* | 103 | 169 | 44 |
| *SSI M vs all other pupfishes* | 1963 | 228 | 158 |
| *SSI S vs all other pupfishes* | 5159 | 58 | 140 |
| *SSI P vs vs all other pupfishes* | 1070 | 573 | 326 |

**Table S5. Top genes with the highest fold-change across craniofacial and caudal tail tissues and across species (Figure 3).** Columns display top DEGs shared between tissues (left), as well as DEGs exclusive to the craniofacial or caudal tissues, per species. Genes that are shared in the same regulation category (either down- or up-regulated) between species are colored (orange for *alox15b* and purple for *nox5* shared between F and Ps, and light-blue for *Serpina1*, which is shared between M and S). F: *C. fontinalis*, NCA: North Carolina *C. variegatus*, SSIA: SSI *C. variegatus*, M: *C. brontotheroides*, S: *C. sp.* ‘wide-mouth’ and P: *C. desquamator.*

|  | Shared DEGs between tissues | | Craniofacial-exclusive DEGs | | Caudal tail-exclusive DEGs | |
| --- | --- | --- | --- | --- | --- | --- |
| Species | Down | Up | Down | Up | Down | Up |
| F | *Alox15b* | Alox15b | Crygm1 | Sept14 | *Nox5* | Cax2 |
| NCA |  |  | Sept5 | Gimap7 | Foxi3a | Igsf10 |
| SSIA |  |  | Mr1 | Laao | Gig3a | Serpina1 |
| M |  |  | Alox15b | Lsm4 | *Serpina1* | Gig2e |
| S |  |  | Crygm3 | Gimap8 | *Serpina1* | B4galt1 |
| P | *Alox15b* | Igsf10 | Sept14 | Olfm4 | *Nox5* | Fto |
